# Human Umbilical Cord Mesenchymal Stem Cell-Derived Extracellular Vesicles Inhibit Endometrial Cancer Cell Proliferation and Migration through Delivery of Exogenous miR-302a

**DOI:** 10.1155/2019/8108576

**Published:** 2019-03-14

**Authors:** Xin Li, Li li Liu, Ju lei Yao, Kai Wang, Hao Ai

**Affiliations:** ^1^Department of Obstetrics and Gynecology, Third Affiliated Hospital of Jinzhou Medical University, Jinzhou, Liaoning 121001, China; ^2^Department of Obstetrics and Gynecology, Dalian Maternity Hospital, Dalian Obstetrics and Gynecology Hospital Affiliated to Dalian Medical University, Dalian, Liaoning 116000, China; ^3^Clinical and Translational Research Center, Shanghai First Maternity and Infant Hospital, Tongji University School of Medicine, Shanghai 200040, China; ^4^Department of Obstetrics and Gynecology, The First Affiliated Hospital, Key laboratory of Follicular Development and Reproductive Health of Liaoning Province, Jinzhou Medical University, Jinzhou, Liaoning 121001, China

## Abstract

MicroRNAs (miRNAs) are potential therapeutic targets in endometrial cancer, but the difficulties associated with their delivery to tumor target cells have hampered their applications. Human umbilical cord mesenchymal stem cells (hUCMSCs) have a well-recognized tumor-homing ability, emphasizing the capacity of tumor-targeted delivery of extracellular vesicles. hUCMSCs release extracellular vesicles rich in miRNAs, which play a vital role in intercellular communication. The purpose of this study was to verify a potential tumor suppressor microRNA, miR-302a, and engineered hUCMSC extracellular vesicles enriched with miR-302a for therapy of endometrial cancer. Here, we observed that miR-302a was significantly downregulated in endometrial cancer tissues when compared with adjacent tissues. Overexpression of miR-302a in endometrial cancer cells robustly suppressed cell proliferation and migration. Meanwhile, the proliferation and migration were significantly inhibited in endometrial cancer cells when cultured with miR-302a-loaded extracellular vesicles derived from hUCMSCs. Importantly, our data showed that engineered extracellular vesicles rich in miR-302 significantly inhibited the expression of cyclin D1 and suppressed AKT signaling pathway in endometrial cancer cells. These results suggested that exogenous miR-302a delivered by hUCMSC-derived extracellular vesicles has exciting potential as an effective anticancer therapy.

## 1. Introduction

Endometrial cancer (EC) is the most common gynecologic malignancy, and the incidence of EC has increased markedly with a higher prevalence in younger women [1]. Endometrioid adenocarcinoma is the most frequently occurring histological type. Increase in the incidence of EC and the lack of powerful yet nontoxic treatment strategies indicate the need of developing novel treatment strategies for this malignancy [2].

MicroRNAs (miRNAs) are a new class of noncoding RNAs and have become an important regulator of several cellular processes [3]. They have the ability to modulate posttranscriptional gene expression to affect cell processes, such as cell proliferation, migration, differentiation, and angiogenesis [4–6]. Based on previous studies, miR-302a is located on chromosomal region 4 which acts as a tumor suppressor in different cancer types, such as human melanoma [7], breast cancer [8], malignant germ cell tumor [9], and cervical carcinoma [10]. miR-302a has also been reported to reduce cell proliferation and tumor formation by downregulating cyclin D1 (CCND1) and AKT1 [10]. Extracellular vesicles (EVs) containing exosomes and microvesicles are important mediators of cell-to-cell communication. They often carry specific molecules like proteins, nucleic acids, and lipids to distant cells, modulating gene expression of the recipient cells [11, 12]. Studies have shown that exosomes were engineered as delivery vehicles which transfer bioactive molecules to cancer cell for treatment [13, 14]. Kamerkar et al. showed that the engineered exosomes carrying anti-KRAS small RNA were used to treat pancreatic cancer [15].

Mesenchymal stem cells (MSCs) are widely studied in regenerative medicine, which have the ability in self-renewing and multipotent differentiation [16, 17]. MSCs can be isolated from human umbilical cord Wharton's jelly and release massive extracellular vesicles like exosomes and microvesicles [18, 19]. Recent studies have shown that MSC-derived EVs can be modified to the carriers of antitumor agents, which have the potential to treat many different types of tumors [20, 21]. It has been proved that EVs derived from MSCs have strong cargo-loading capacity which is emerging as a cell-free therapy in tumor.

In this study, we hypothesized that human umbilical cord mesenchymal stem cell- (hUCMSC-) derived EV-delivered miR-302a might play a significant role in the suppression of EC cell proliferation and migration. We investigated the role of miR-302a and miR-302a-enriched EVs derived from hUCMSCs in EC cell proliferation and migration and the underlying mechanisms. These studies suggested that exogenous miR-302a delivered by hUCMSC-derived EVs has exciting potential as an effective anticancer therapy.

## 2. Materials and Methods

### 2.1. Isolation of hUCMSCs

The procedure was approved by the research ethics committee of Shanghai First Maternity and Infant Hospital. Umbilical cord samples were collected from full-term births after normal or cesarean delivery, then rinsed in phosphate-buffered saline (PBS; HyClone, Logan, UT) to remove blood vessels and cut into small pieces in Hank's buffered salt solution (HBSS; Gibco, Grand Island, NY). The pieces of umbilical cord Wharton's jelly were transferred to T-75 flasks with a 10 ml *α*-MEM (Gibco) medium containing 10% fetal bovine serum (FBS; Gibco) and 1% penicillin/streptomycin (P/S; Gibco) and incubated at 37°C in 5% CO_2_. The medium was changed every third day. Cells were trypsinized and passaged at 80–90% confluence.

### 2.2. Identification of hUCMSCs by Flow Cytometry

Detection of hUCMSC surface markers by a FACSCalibur flow cytometer (BD Biosciences, San Jose, CA) is performed as follows. Cells about 1 × 10^6^ were trypsinized and suspended in 100 *μ*l PBS. Then, monoclonal antibodies conjugated with CD45-PE, CD73-APC, CD90-FITC, and CD105-PE-Cy7 (BD Biosciences) were added and incubated for 30 minutes.

### 2.3. Osteogenic Differentiation

Bone differentiation of hUCMSCs was carried out by culturing in differentiation-conditioned medium (Gibco) for 2-3 weeks. Cells of the third passages were cultured in a six-well plate, reaching to 80-90% confluence; then, the medium was replaced with differentiation medium every third day. Cells were washed with PBS and fixed with paraformaldehyde at 4% for 15 minutes. Next, fixed cells were stained with alizarin red S (Sigma-Aldrich, St. Louis, MO) for 15 minutes. Cells were observed by microscopy (Nikon Eclipse Ti, Tokyo, Japan).

### 2.4. Culture of ISK Cells and ECC-1 Cells

Human endometrial cancer cell lines ISK and ECC-1 used in this study were maintained in our laboratory, which were, respectively, cultured in Dulbecco's modified Eagle's medium (DMEM)/F12 medium (Gibco) and Roswell Park Memorial Institute medium (RPMI; Gibco) at 37°C with 5% CO_2_. All media were supplemented with 10% FBS and 1% P/S.

### 2.5. Cell Transfection

We established miR-302a-overexpressing cells in hUCMSC, ISK, and ECC-1 cells using lentivirus transfection. Lentiviral packaging was performed by cotransfection of human 293T cells with pLKO.1 and pLKO.1-miR-302a using Lipofectamine 2000 (Invitrogen, Carlsbad, CA). Lentiviruses were harvested after transfection. hUCMSCs, ISK, and ECC-1 cells were transduced with lentiviruses expressing miR-302a or nonexpressing controls for 24 h in the presence of 8 ng/ml polybrene (Sigma). Medium containing puromycin (10 *μ*g/ml; Invitrogen) was used to select stably transduced cells.

### 2.6. Isolation of Extracellular Vesicles

Extracellular vesicles were isolated from cell culture media of hUCMSCs that were grown in *α*-MEM supplemented with 10% FBS and 1% P/S until the cells reach to 80-90% confluence, and hUCMSCs are cultured in serum-free media for 48 hs. Culture media were collected and centrifuged at 3000 g for 15 minutes at 4°C to remove cellular debris. Extracellular vesicles were isolated from the hUCMSC cultures using the ExoQuick-TC kit (System Biosciences, Palo Alto, CA) according to the manufacturer' s protocol. The extracellular vesicle pellets were resuspended in PBS and stored at -80°C for further use.

### 2.7. Transmission Electron Microscopy

The morphology of the extracellular vesicles was observed using transmission electron microscopy (TEM, Thermo Fisher Scientific, Waltham, MA). 15 *μ*l of extracellular vesicle suspension was fixed on a continuous grid and then negatively stained with 2% aqueous uranyl acetate solution for 1 min. Grids were allowed to thoroughly dry before viewing. The extracellular vesicles were visualized using an FEI Tecnai G2 Spirit transmission electron microscope (Thermo Fisher Scientific) at an acceleration voltage of 120 kV.

### 2.8. Nanoparticle Tracking Analysis for Extracellular Vesicles

ZetaView (Particle Metrix, Meerbusch, Germany) was used to detect nanoparticles of the size and distribution or concentration of extracellular vesicles as recommended by the company. The data were analyzed using the instrument software, ZetaView 8.02.28.

### 2.9. Confocal Microscopy

Purified extracellular vesicles were labeled with the CM-Dil membrane dye (Thermo Fisher Scientific). The extracellular vesicles were stained with 1 *μ*M of CM-Dil and incubated for 15 minutes at 37°C. The extracellular vesicles stained with CM-Dil were collected using the ExoQuick-TC, then rinsed with PBS 2 times and resuspended in 1 ml cell medium (with 1% P/S). ISK cells and ECC-1 cells (5000 cells/well) were incubated with CM-Dil-labeled extracellular vesicles for 16 h at 37°C with 5% CO_2_. Then, the cells were fixed with paraformaldehyde at 4% for 15 minutes and were stained with phalloidin-FITC (Sigma). Nuclei were stained with DAPI (Sigma). The cellular uptake of extracellular vesicles was tested by confocal microscopy (TCS SP8; Leica, Wetzlar, Germany).

### 2.10. RNA Extraction and Quantitative RT-PCR

Total RNA was extracted from hUCMSCs, ISK, and ECC-1 cells using TRIzol reagent (Invitrogen). miRNAs extracted from extracellular vesicles using miRNeasy (Qiagen, Hilden, Germany). RNA concentration was assessed by a NanoDrop 2000c spectrophotometer (NanoDrop Technologies, Wilmington, DE). Reverse transcription reactions were performed with miRcute plus miRNA first-strand cDNA synthesis kit (Tiangen, Beijing, China) for miRNA analysis, and RNA reverse transcription kit (TaKaRa, Dalian, China) was used for mRNA analysis according to the protocol. Quantification of miRNA expression was used by miRcute Plus miRNA qPCR detection kit (Tiangen) on the StepOnePlus PCR system (Thermo Fisher Scientific). Quantitative RT-PCR (qRT-PCR) of mRNA was performed with SYBR Premix Ex Taq (TaKaRa). Expression data of mRNA and miRNA were normalized to *β*-actin and U6 reference genes, respectively, using the 2-ΔΔCT method. PCR conditions consisted of denaturation at 95°C for 30 seconds and 40 cycles of 95°C for 15 seconds and annealing at 60°C for 20 seconds. The primer sequences were as follows: *β*-actin forward primer: 5′-AACTCCATC ATGAAGTGTGACG-3′ and *β*-actin reverse primer: 5′-GATCCACATCTGCTGGAAGG-3′; cyclin D1 forward primer: 5′-GCTGCGAAG TGGAAACCATC-3′ and cyclin D1 reverse primer: 5′-CCTCCTTC TGCACACATTTGAA-3′; and AKT forward primer: 5′-AGCGACGTGGCTATTGTGAAG-3′ and AKT reverse primer: 5′-GCCATCATTCTTGAGGAGGAAGT-3′. The primers of miR-302a and U6 were obtained from RiboBio Company (Guangzhou, China).

### 2.11. Western Blot Analyses

The concentration of protein was quantified using BCA protein assay kit (Thermo Fisher Scientific). Proteins were extracted using a lysis buffer (RIPA, Beyotime, Shanghai, China) containing protease inhibitors. A total of 20 *μ*g protein was separated on SDS-PAGE gel and transferred to a polyvinylidene fluoride (PVDF, Roche, Mannheim, Germany) membrane that was blocked for 2 h at room temperature with 5% bovine serum albumin (BSA). Blots were probed with the following antibodies: CD 81 (1 : 1000, System Biosciences), HSP 70 (1 : 1000, System Biosciences), cyclin D1 (1 : 1000, Abcam), AKT, p-AKT (1 : 1000, Cell Signaling Technology (CST), Danvers, MA), and *β*-actin (1 : 1000, Abcam) at 4°C overnight. The next morning, membranes were incubated with anti-rabbit IgG antibody (1 : 5000, CST). The immunoreactive bands were detected with chemiluminescence detection kit (MilliporeSigma, Burlington, MA) and visualized using the FluorChem E imaging instrument (ProteinSimple, San Jose, CA).

### 2.12. Cell Proliferation

Cell proliferation was measured by MTS (Promega, Madison, WI). ISK/ECC-1 cells (2 × 10^3^ cells/well) were cultured in a medium containing 10% FBS in 96-well plates for 48 hs. ISK/ECC-1 cells were seeded in 96-well plates (2 × 10^3^ cells/well) with complete medium (10% FBS). The cells were starved for 8 h after attachment and incubated with 100 *μ*g/ml extracellular vesicles for 48 hs. Then, 20 *μ*l MTS was added to each well and incubated for 1 h before the plates were analyzed with a microplate spectrophotometer (Thermo Fisher Scientific).

### 2.13. Transwell Migration Assay

The migration ability of cells was examined using a 24-well transwell insert system with a pore size of 8 *μ*m (Costar; Corning, NY). Briefly, ISK/ECC-1 cells (5 × 10^4^ cells/well) were cultured in a medium containing 2% FBS in the upper chambers of the inserts for 16 h. The bottom wells of the chamber were filled with a medium with 10% FBS prior to cell seeding. ISK/ECC-1 cells (1 × 10^5^ cells/well) were plated on the upper chambers of the insert, which contained a 200 ml medium with 1% P/S. The lower chambers were filled with the 800 ml conditioned medium including 100 *μ*g/ml extracellular vesicles, 2% extracellular vesicle-depleted FBS (System Biosciences), and 1% P/S. After 16 h in culture, calcein AM (0.2 *μ*g/ml, Invitrogen) was added to each chamber and incubated for 30 minutes. The labeled cells were observed and photographed with a Nikon Eclipse Ti fluorescence microscope (Tokyo, Japan).

### 2.14. Immunohistochemistry (IHC)

Tissue was paraffin-embedded and sliced into 4 *μ*m thick sections. Next, sections were paraffinized, rehydrated, and boiled at 100°C for 20 minutes to antigen retrieval. The sections were incubated with rabbit anti-human monoclonal cyclin D1 antibody (1 : 1000, Abcam) overnight at 4°C and goat anti-rabbit HRP secondary antibody (1 : 500, Abcam) 37°C for 30 minutes. Finally, the sections were stained with 3,3′-diaminobenzidine and then counterstained with hematoxylin.

### 2.15. Statistical Analysis

All experiments were performed in triplicate. Data are presented as the mean ± SEM. The statistical significance was tested using ANOVA, and comparisons between two groups were carried out with an independent *t*-test. *P* < 0.05 was considered to be statistically significant, and *P* < 0.01 was considered to be very significant. All statistical analyses were performed using GraphPad Prism 6 (GraphPad Software Inc., La Jolla, CA).

## 3. Results

### 3.1. Characterization of hUCMSCs and Extracellular Vesicles

**Figure 1:**
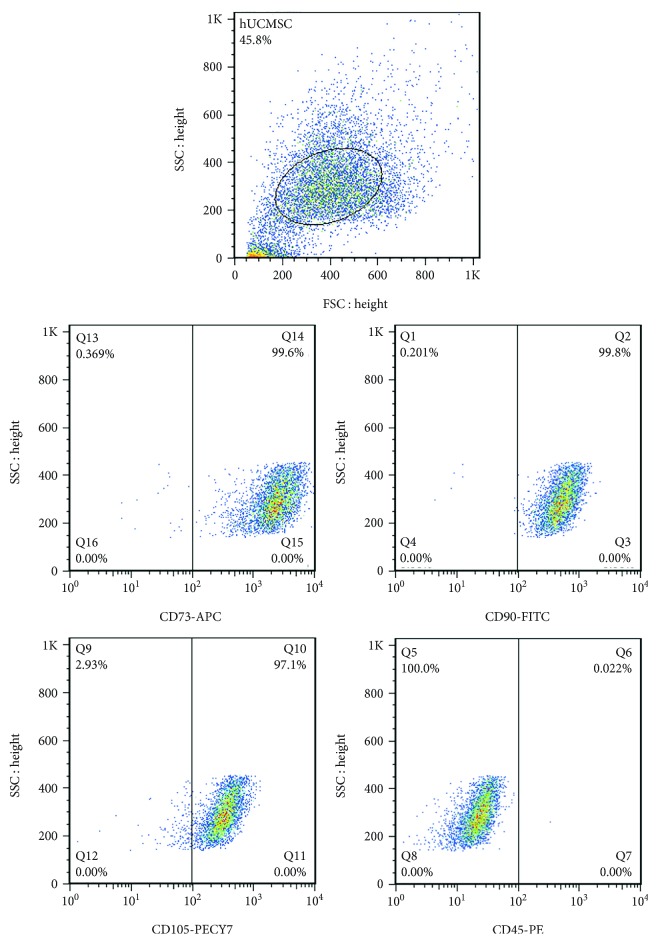
Identification of hUCMSCs by flow cytometry for CD90, CD73, CD105 (positive markers), and CD45 (negative marker).

**Figure 2:**
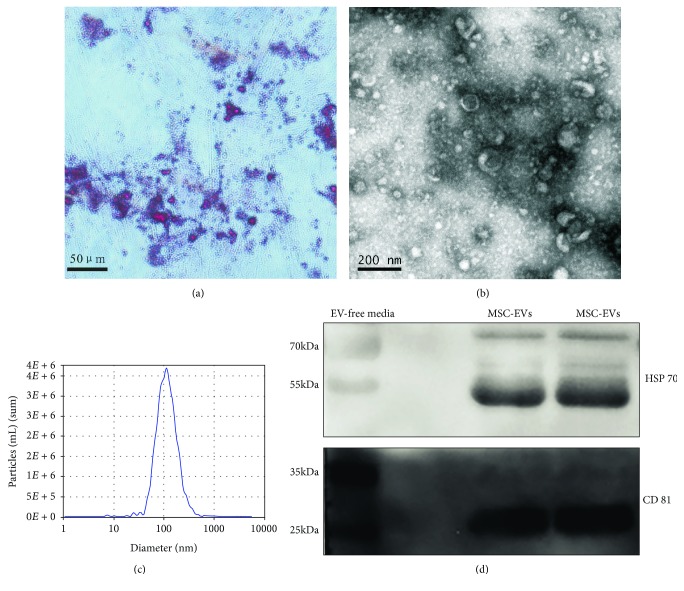
Osteogenic differentiation and characteristics of extracellular vesicles collected from the media of hUCMSCs. (a) Osteogenic differentiation of hUCMSCs stained positive with alizarin red S. (b) Transmission electron microscopy analysis of extracellular vesicles secreted by hUCMSCs. (c) NTA profile of extracellular vesicles derived from hUCMSCs. (d) CD 81 and HSP 70 (common extracellular vesicle markers) immunoblots of extracellular vesicles derived from hUCMSCs.

We successfully isolated hUCMSCs from human umbilical cord Wharton's jelly using a tissue adherence method. These hUCMSCs express mesenchymal antigens, which are positive for CD73, CD90, and CD105, while lacking hematopoietic marker CD45 (Figure 1). We used the third generation of hUCMSCs to perform osteogenic differentiation, stained with alizarin red S for positive detection (Figure 2(a)). We used ExoQuick-TC kit to isolate EVs from the culture medium of hUCMSCs, then visualized by transmission electron microscopy. The results showed typical rounded particles ranging from 40-200 nm in diameter (Figure 2(b)). Nanoparticle tracking analysis (NTA) characterized the size of EVs derived from hUCMSCs at a peak of 108 nm (Figure 2(c)). Western blotting analysis demonstrated the presence of EV markers such as HSP 70 and CD 81 (Figure 2(d)).

### 3.2. Overexpression of miR-302a Inhibits EC Cell Growth *In Vitro*

**Figure 3:**
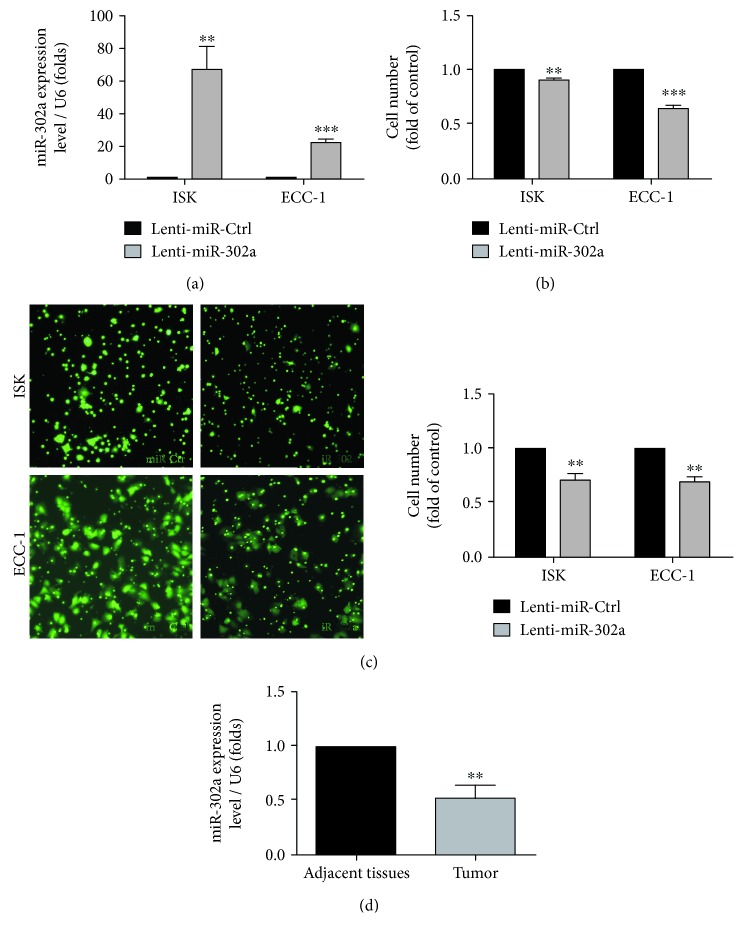
Overexpression of miR-302a inhibits the proliferation and migration of EC cells *in vitro*. (a) The efficiency of overexpression of miR-302a was determined by quantitative RT-PCR analyses. (^∗∗^*P* < 0.01, ^∗∗∗^*P* < 0.001). (b) Effects of overexpression of miR-302a on the proliferation of ISK and ECC-1 cells were measured by MTS (^∗∗^*P* < 0.01, ^∗∗∗^*P* < 0.001). (c) Effects of overexpression of miR-302a on the migration of ISK and ECC-1 cells were measured using the transwell migration assay. The fold change in the cell number relative to the control from three independent experiments (^∗∗^*P* < 0.01). (d) Expression levels of miR-302a in adjacent tissues (*n* = 7) and EC tissues (*n* = 7) were determined by quantitative RT-PCR (^∗∗^*P* < 0.01).

To investigate the impact of miR-302a on ISK and ECC-1 EC cell proliferation and migration, we stably overexpressed miR-302a in these cell lines using a lentiviral expression vector. Our results displayed that miR-302a was highly expressed in ISK and ECC-1 cells after being transfected with lentivirus (Figure 3(a)). The proliferation and migration capacity of ISK and ECC-1 cells was significantly suppressed when miR-302a was overexpressed in these cells (Figures 3(b) and 3(c)). Meanwhile, miR-302a expression in EC tissues (*n* = 7) and adjacent tissues (*n* = 7) was measured by quantitative RT-PCR. Our data showed that miR-302a expression was downregulated in EC tissues compared with adjacent tissues (Figure 3(d)).

### 3.3. Enrichment of miR-302a in hUCMSCs and hUCMSC-Derived Extracellular Vesicles

**Figure 4:**
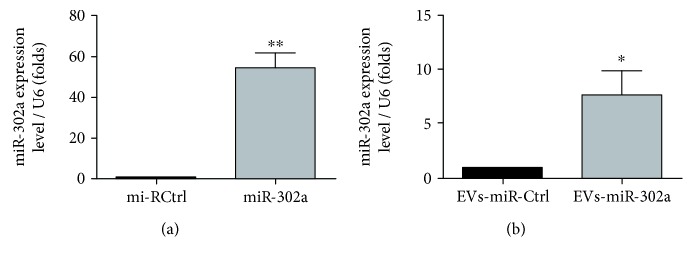
Enrichment of miR-302a in hUCMSCs and extracellular vesicles. (a, b) The efficiency of overexpression of miR-302a in hUCMSCs and hUCMSC-derived extracellular vesicles was determined by quantitative RT-PCR analyses (^∗∗^*P* < 0.01) (^∗^*P* < 0.05).

To investigate the role of miR-302a on intercellular communication by EVs, we collected the culture media to gain the EVs rich in miR-302a. The results showed that miR-302a in hUCMSCs was remarkably overexpressed after infection with lentivirus (Figure 4(a)). And miR-302a in EVs derived from hUCMSCs showed a significant increase by quantitative RT-PCR (Figure 4(b)).

**Figure 5:**
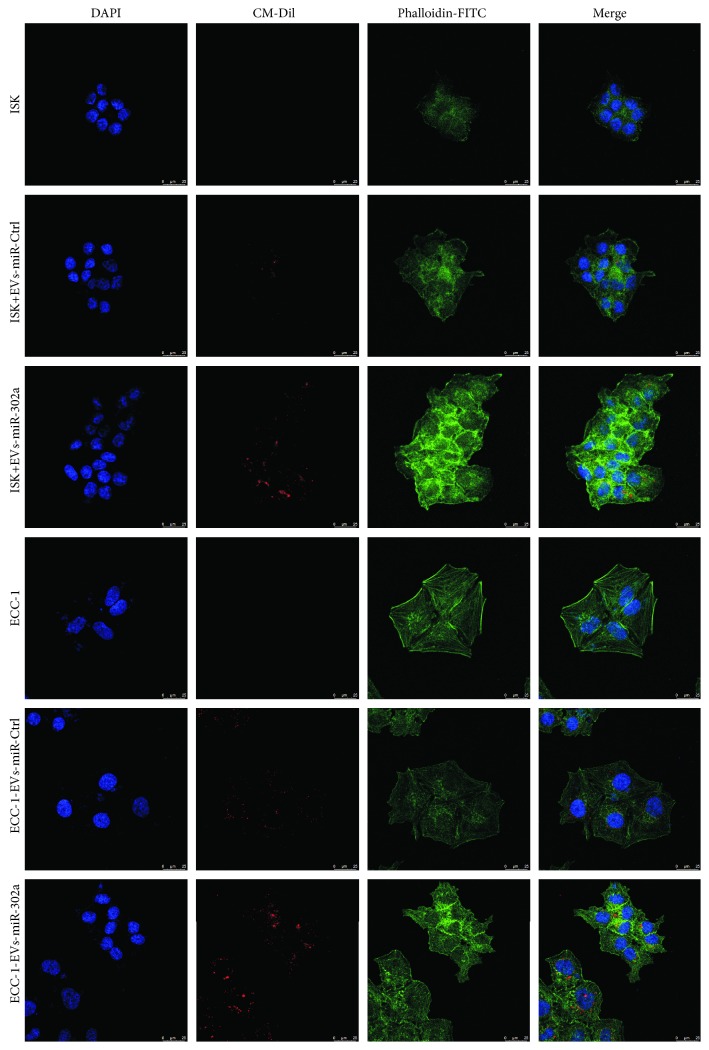
Cellular internalization of hUCMSC-derived extracellular vesicles into EC cells ISK and ECC-1 cells incubated with CM-Dil-labeled extracellular vesicles (100 *μ*g/ml) or negative controls without extracellular vesicles for 16 h (CM-Dil in red, phalloidin-FITC in green, and DAPI in blue).

To validate whether ISK and ECC-1 cells could internalize miR-302a-abundant EVs secreted from hUCMSCs, EVs labeled with CM-Dil (red) were incubated with ISK and ECC-1 cells for 16 h. Our results demonstrated that ISK and ECC-1 cells marked with phalloidin-FITC (green) could internalize EVs labeled by CM-Dil (Figure 5). These findings suggested that EVs which contained miR-302a derived from hUCMSCs could be delivered to EC cells.

### 3.4. Extracellular Vesicles Rich in miR-302a Inhibit EC Cell Growth *In Vitro*

**Figure 6:**
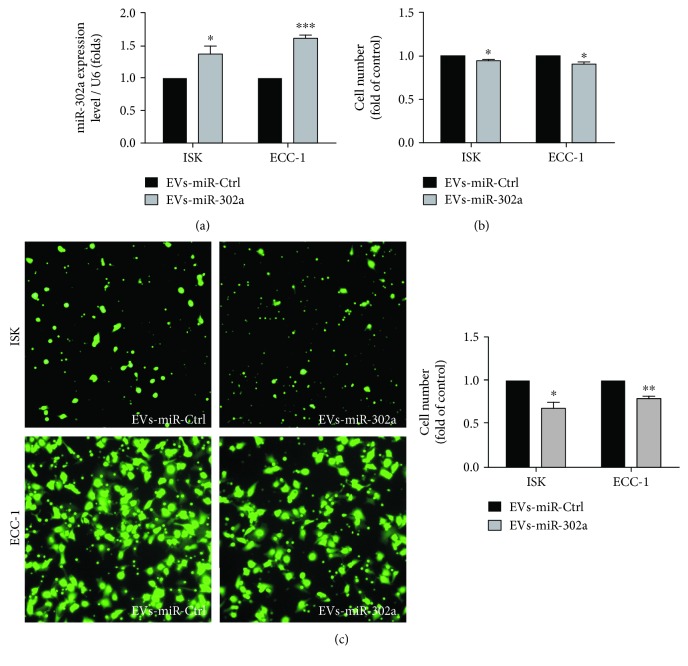
Extracellular vesicle-loaded miR-302a inhibits EC cell proliferation and migration. (a) The expression of miR-302a in ISK and ECC-1 cells after incubation of extracellular vesicle-loaded miR-302a was determined by quantitative RT-PCR analyses (^∗^*P* < 0.05, ^∗∗∗^*P* < 0.001). (b) The proliferation capacity of ISK and ECC-1 cells treated with extracellular vesicle-loaded miR-302a was measured by MTS (^∗^*P* < 0.05). (c) The migration capacity of ISK and ECC-1 cells incubated with extracellular vesicles rich in miR-302a was detected using the transwell migration assay. The number of migrated cells was counted, and the data is suggested as the fold change from the control of three independent experiments (^∗^*P* < 0.05, ^∗∗^*P* < 0.01).

To explore whether miR-302a delivered by hUCMSC-derived EVs has the potential impact on EC, we detected cell migration and proliferation of ISK and ECC-1 after treatment with miR-302a-enriched EVs. The expression level of miR-302a in ISK and ECC-1 cells was upregulated after incubation of miR-302a-enriched EVs compared with miR-Ctrol-enriched EVs (Figure 6(a)). Meanwhile, treatment with miR-302a-abundant EVs caused a significant inhibition of ISK and ECC-1 cell proliferation and migration (Figures 6(b) and 6(c)).

### 3.5. Extracellular Vesicles Rich in miR-302a Inhibit Cyclin D1 and AKT Expression in EC Cells

**Figure 7:**
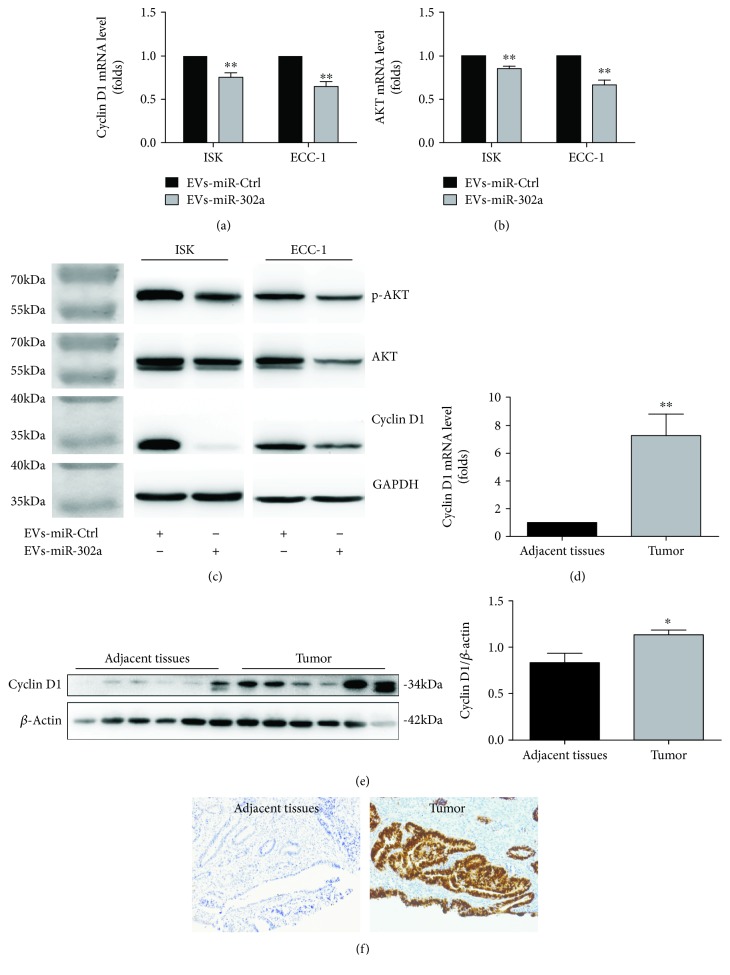
Mechanism of action of miR-302a. (a, b) Expression levels of cyclin D1 and AKT in ISK and ECC-1 cells after indicated treatment was measured by quantitative RT-PCR analyses (^∗∗^*P* < 0.01). (c) Expression levels of cyclin D1, AKT, and p-AKT in ISK and ECC-1 cells after indicated treatment was determined by western blot analyses. (d, e) The mRNA and protein expression of cyclin D1 in endometrial cancer tissues (*n* = 6) and adjacent tissues (*n* = 6) (^∗^*P* < 0.05, ^∗∗^*P* < 0.01). (f) Representative image of cyclin D1 expression in EC samples and adjacent tissues was determined by IHC.

It is well studied that miR-302a was involved in the regulation of cell growth by regulation of cyclin D1 and AKT [10]. Indeed, our results also indicated that the mRNA expression of cyclin D1 and AKT was significantly reduced after incubation with miR-302a-enriched EVs (Figures 7(a) and 7(b)). Similarly, the protein levels of cyclin D1 and AKT and the phosphorylation of AKT were downregulated in both ISK and ECC-1 cells when treated with miR-302a-loaded EVs (Figure 7(c)). Moreover, the mRNA and protein expression of cyclin D1 was upregulated in EC tissues compared with adjacent tissues (Figures 7(d) and 7(e)). Finally, the IHC analysis also confirmed that cyclin D1 protein staining was significantly positive in EC tissues (Figure 7(f)).

## 4. Discussion

In the present study, we engineered miR-302a-loaded EVs derived from hUCMSCs and demonstrated that these miR-302a-loaded EVs could significantly inhibit the cell proliferation and migration through reduction of cyclin D1 expression and suppression of AKT signaling pathway in endometrial cancer cells. These results implied that exogenous miR-302a delivered by hUCMSC-derived EVs has exciting potential as an effective anticancer therapy.

A growing number of studies have shown that miRNAs can be utilized as a potential molecular target for tumor therapy [22]. Human miR-302 cluster contains miR-302a, miR-302b, miR-302c, miR-302d, and miR-367, which plays an important role in biological processes and regulates many pathological changes, including cancer growth and development [23]. In this study, the expression level of miR-302a in EC tissues was significantly lower than that in adjacent tissues, suggesting that it acted a pivotal role in EC development. Indeed, miR-302a has been reported to be downregulated in malignant cells of gastric cancer, breast cancer, ovarian cancer, and colorectal cancer and plays a key role in the regulation of growth and metastasis of cancer cells [24, 25]. Moreover, in the present study, overexpression of miR-302a inhibited the proliferation and migration of EC cells, indicating miR-302a as a potential tumor suppressor, which is consistent with other studies [26]. For example, miR-302 inhibits proliferation of cells and induces apoptosis of cells in human melanoma [7], breast cancer [8], malignant germ cell tumor [9], and cervical carcinoma [10].

Vehicles for delivery of therapeutic miRNAs, such as liposomes and viral vectors, are limited because of the insecurity and low efficiency. EVs are gaining increasing popularity as potential therapeutic agents, according to the fact that EVs including exosomes have the ability to transfer genetic materials (miRNA) between different cell types [27–30]. We successfully engineered hUCMSCs to secrete EVs enriched with miR-302a and testified the potential therapeutic impacts of these EVs on EC cells *in vitro*. These results support the view that hUCMSCs are efficient EV producers that can be manufactured in culture in a large scale for cell-free therapy [31].

MSCs as delivery vehicles have been well studied [32, 33], while the use of EVs to carry miRNAs in EC is relatively unexplored. In this context, we showed that treatment with miR-302a-enriched EVs derived from hUCMSCs inhibited proliferation and migration of EC cells *in vitro*. These results support the notion that EVs are capable of transferring functional miRNA to cancer cells. Further, we found that the expression level of cyclin D1 in EC tissues was higher than that in adjacent tissues, in which the enhancement of cyclin D1 expression was also identified in many different types of cancers [34–36]. Indeed, our work demonstrated that miR-302a was a potent tumor suppressor in EC, the effect of which was mediated in part by regulation of cyclin D1. Treatment with EVs rich in miR-302a suppressed cyclin D1, one of the known direct targets of miR-302a, and reduced expression of AKT and phosphorylation of AKT. It is widely studied that phosphatidylinositol-3 kinase (PI3K)/AKT pathway is activated in a variety of malignancies, such as EC, breast cancer, and ovarian cancer, which leads to cell proliferation and growth by regulating the cyclin D1 level [37–39]. Overall, our results provided strong evidence that miR-302a-enriched EVs caused a significant reduction in proliferation and migration of EC cells by downregulating cyclin D1 through an interaction with AKT, a cell cycle regulator.

In this study, we have shown that it is possible for hUCMSCs to engineer a large number of bioactive EVs, which attain targeted miRNAs to delivery. Besides, hUCMSC-derived EVs represent the tumor-homing capability of a parent cell which has a tendency to target a tumor. We believe that EVs rich in miR-302a may be an effective anticancer therapy in EC.

## Data Availability

All data included in this study are available upon request by contact with the corresponding author.
